# RasGRF1 regulates proliferation and metastatic behavior of human alveolar rhabdomyosarcomas

**DOI:** 10.3892/ijo.2012.1536

**Published:** 2012-06-28

**Authors:** MACIEJ TARNOWSKI, GABRIELA SCHNEIDER, GABRIELE AMANN, GEOFFREY CLARK, PETER HOUGHTON, FREDERIC G. BARR, LUKAS KENNER, MARIUSZ Z. RATAJCZAK, MAGDA KUCIA

**Affiliations:** 1 Stem Cell Institute at James Graham Brown Cancer Center, University of Louisville, Louisville, KY, USA;; 2 Department of Physiology Pomeranian Medical University, Szczecin, Poland;; 3 Clinical Institute of Pathology, Medical University of Vienna, Vienna, Austria;; 4 World Children’s Cancer Center, Columbus, OH, USA;; 5 Ludwig Boltzmann Institute for Cancer Research, Vienna, Austria;; 6 Department of Pathology and Laboratory Medicine, University of Pennsylvania School of Medicine, Philadelphia, PA, USA

**Keywords:** rhabdomyosarcoma, Ras, RasGRF1, metastasis

## Abstract

The involvement of the Ras superfamily of GTPases in the pathogenesis of rhabdomysarcoma (RMS) is not well understood. While mutant H-Ras leads to embryonal RMS (ERMS) formation in experimental animals and in Costello syndrome patients, no data exists on the potential role of Ras GTPases in the pathogenesis of alveolar RMS (ARMS). To address this issue better, we focused on the role of the GTP exchange factor RasGRF1 in this process. We observed that, in comparison to normal skeletal muscle cells, RasGRF1 mRNA is upregulated in the majority of human ARMS cell lines and subsequently confirmed its high expression in patient samples. By employing confocal microscopy analysis, we observed RasGRF1 accumulation in cell filopodia, which suggests its involvement in ARMS cell migration. Furthermore, we observed that RasGRF1 becomes phosphorylated in ARMS after stimulation by several pro-metastatic factors, such as SDF-1 and HGF/SF, as well as after exposure to growth-promoting Igf-2 and insulin. More importantly, activation of RasGRF1 expression correlated with activation of p42/44 MAPK and AKT. When the expression of RasGRF1 was down-regulated in ARMS cells by an shRNA strategy, these RasGRF1-kd RMS cells did not respond to stimulation by SDF-1, HGF/SF, Igf-2 or insulin by phosphorylation of p42/44 MAPK and AKT and lost their chemotactic responsiveness; however, their adhesion was not affected. We also observed that RasGRF1-kd ARMS cells proliferated at a very low rate *in vitro*, and, more importantly, after inoculation into immunodeficient SCID/beige inbred mice they formed significantly smaller tumors. We conclude that RasGRF1 plays an important role in ARMS pathogenesis and is a new potential therapeutic target to inhibit ARMS growth.

## Introduction

Rhabdomyosarcoma (RMS) is the most common soft-tissue sarcoma of adolescence and childhood, accounting for 5% of all malignant tumors in patients under 15 years of age. Most RMS tumors originate in the head and neck region, urogenital tract, and extremities (1–6). Based on histology, there are two major subtypes of RMS: alveolar (A)RMS and embryonal (E) RMS (7). Clinical evidence indicates that ARMS is more aggressive and has a significantly worse outcome than ERMS. Genetic characterization of RMS has identified markers that show excellent correlation with histologic subtype. Specifically, ARMS is characterized by the translocation t(2;13)(q35;q14) in 70% of cases and the variant translocation t(1;13)(p36;q14) in a smaller percentage of cases. These translocations generate *PAX3-FOXO1* and *PAX7-FOXO1* fusion genes that encode the fusion proteins PAX3-FOXO1 and PAX7-FOXO1, which are believed to act in cell survival and deregulation of the cell cycle in ARMS cells. Evidence accumulates that ARMS and ERMS are two different disorders. While ARMS may originate from primitive uncommitted mesodermal cells, ERMS originates probably from more differentiated myoblasts (8). This interesting concept however, needs more evidence.

As with other malignancies, the major clinical problem with RMS is its tendency to metastasize and infiltrate various organs. This process is directed by several chemokines, such as stromal-derived factor-1 (SDF-1), interferon-inducible T-cell alpha chemoattractant (I-TAC), and hepatocyte growth factor/scatter factor (HGF/SF). In addition, the family of insulin factors, including insulin (Ins), insulin-like growth factor-1 (Igf-1), and insulin-like growth factor-2 (Igf-2), plays an important role both in stimulating proliferation and migration of RMS cells (9–12). In addition to *PAX-FOXO1* fusion genes, aberrant expression of p53, p16^INK4A^/p14^ARF^, and activation of the H-Ras pathway have been postulated to function in RMS pathogenesis (13).

The Ras superfamily of guanosine triphosphatases (GTPases), which includes H-, K-, and N-Ras and other closely related isoforms, are regulated switches that control many intra-cellular pathways associated with the control of cell proliferation and migration (14–16). The Ras GTPases act by cycling between guanosine triphosphate (GTP)-bound states that can couple to downstream events and guanosine diphosphate (GDP)-bound states that do not activate those events (16). The conversion between these states is governed by several groups of enzymes, including GTP-exchange factors (GEFs), which catalyze the release of GDP and subsequent binding of GTP to activate these proteins, and GTPase-activating proteins (GAPs), which greatly stimulate the endogenous GTPase activity of Ras proteins and thereby stimulate their inactivation.

The potential role of Ras pathway activation is demonstrated very well for ERMS but not for ARMS cases. To support this role, it has been demonstrated in a zebrafish model that expression of mutant H-Ras induced ERMS tumors by day 10 of life (17). Furthermore, ERMS has been reported in Neurofibromatosis type 1 (18,19), Noonan syndrome (20,21) and Costello syndrome patients (22–24) with increased Ras signaling cascade caused by mutation in one of several genes encoding proteins in this pathway - a phenomenon known in the literature as ‘RASopathies’ (25). In sporadic RMS tumors, Ras family mutations have been found in about 20% of ERMS but not in any ARMS cases. Since the combination of Ras activation along with expression of dominant-negative p53 or SV40 early region proteins and PAX-FOXO1 in murine mesenchymal stem cells (MSCs) leads to formation of ARMS-like tumor cells, we became interested in a potential role of Ras signaling in the pathogenesis of ARMS. Because no Ras mutations have been reported in ARMS patients, we hypothesized that RasGRF1 (or CDC25^Mm^) which is a GTP exchange factor for Ras GTPases, plays a role in the pathogenesis of ARMS.

In addition, it was another reason why we become interested in a potential role of RasGRF1 in pathogenesis of ARMS. Namely, as it has been postulated this type of RMS develops in some primitive uncommitted mesodermal cell (8,26). On other hand RasGRF1 plays an important role in the development of primitive very small embryonic-like stem cells (VSELs) residing in adult tissues (27) as demonstrated in a recent elegant study are precursors for the mesodermal and mesenchymal stem cells (19). Therefore, based on this and other studies (28,29) RMS could develop in stem cells related to mesenchymal lineage. To support further this hypothesis, the analysis of epigenetic changes in VSELs identified unique methylation patterns of differentially methylated regions (DMRs) in several imprinted genes including RasGRF1, Igf2-H19 and KCNQ1 that explain dormant state of VSELs in adult tissues (27). At the same time, the same genes (Igf2 and KCNQ1) due to epigenetic changes at their DMR loci are overexpressed in rapidly proliferating RMS cells. This involvement of imprinted genes in pathogenesis of RMS explains why we become interested to see if RasGRF1 similarly as Igf2 and KCNQ1 could be also involved in pathogenesis of ARMS.

In this study, our findings indicate that RasGRF1 mediates the chemotactic responsiveness of RMS cells to SDF-1, HGF/SF, Igf-1, and Ins. Furthermore, knockdown of RasGRF1 in RMS cells inhibited ARMS cell growth *in vitro* and tumor formation *in vivo* in immunodeficient mice. We therefore postulate that RasGRF1 plays an important role in ARMS pathogenesis and is a new potential target to inhibit ARMS growth.

## Materials and methods

### Animals

This study was performed in accordance with the guidelines of the Animal Care and Use Committee of the University of Louisville School of Medicine and with the Guide for the Care and Use of Laboratory Animals (Department of Health and Human Services, publication no. NIH 86-23).

### Cell lines

We used six human ARMS cell lines (gift of Dr Peter Houghton, Nationwide Children’s Research Hospital, Columbus, OH) (RH2, RH4, RH18, RH28, RH30 and RH41). RMS cells used for experiments were cultured in Roswell Park Memorial Institute medium (RPMI)-1640 (Sigma, St. Louis, MO), supplemented with 100 IU/ml penicillin, 10 *μ*g/ml streptomycin, and 50 *μ*g/ml neomycin (Life Technologies, Inc., Grand Island, NY) in the presence of 10% heat-inactivated fetal bovine serum (FBS, Life Technologies). The cells were cultured in a humidified atmosphere at 37°C in 5% CO_2_ at an initial cell density of 2.5×10^4^ cells/flask (Corning, Cambridge, MA) and the media were changed every 48 h.

### Real-time quantitative reverse transcription PCR (RQ-PCR)

Total RNA was isolated from cells treated with hypoxia and from controls with the RNeasy Kit (Qiagen, Valencia, CA). The RNA was reverse-transcribed with MultiScribe reverse transcriptase and oligo-dT primers (Applied Biosystems, Foster City, CA). Quantitative assessment of mRNA levels was performed by real-time RT-PCR on an ABI 7500 instrument with Power SYBR Green PCR Master Mix reagent. Real-time conditions were as follows: 95°C (15 sec), 40 cycles at 95°C (15 sec), and 60°C (1 min). According to melting point analysis, only one PCR product was amplified under these conditions. The relative quantity of a target, normalized to the endogenous control β-2 microglobulin gene and relative to a calibrator, is expressed as 2-ΔΔCt (-fold difference), where Ct is the threshold cycle, ΔCt = (Ct of target genes) - (Ct of endogenous control gene, β-2 microglobulin), and ΔΔCt = (ΔCt of samples for target gene) - (ΔCt of calibrator for the target gene). The following primer pairs were used: RasGRF1 F: 5′-GCCACCAATCGTGTCTTGAA-3′; RasGRF1 R: 5′-CAAAGTCCTGAGAGTGCTTGGA-3′.

### Immunohistochemistry

Formalin-fixed, paraffin-embedded sections (4 *μ*m) were stained for RasGRF1. The de-waxed, rehydrated sections were heated in 0.01 M citrate buffer at pH 6.0 in an autoclave. Afterwards the endogenous peroxidase activity was blocked in 3% hydrogen peroxide in PBS for 10 min. After washing the blocking was performed using the avidin/biotin blocking kit (Vector Laboratories, Cambridge, UK). The sections were incubated with the primary antibody targeting RasGRF1 (Santa Cruz Biotechnology, Santa Cruz, CA, USA) in 1:200 dilution at 4°C overnight. The immunohistochemistry detection was done with the IDetect Super Stain System HRP (ID Laboratories, London, ON, Canada). The signal was visualized with 3-amino-9-ethylcarbazole (ID Laboratories). Afterwards the sections were counterstained with hematoxylin.

The immunohistochemical staining on 4 samples was analyzed with the software HistoQuest™ from TissueGnostics GmbH (Vienna, Austria, www.tissuegnostics.com). The HistoQuest™ software permits quantification of expression intensities on immunostained slides. The results are visualized in scattergrams and/or histograms. Images were taken with a Zeiss AxioImager Z.1 microscope. Statistical analysis of the data was performed using the t-test (Graph Pad Prism Software, San Diego, CA, USA).

### Knockdown of RASGRF1 with short hairpin RNA

In RNAi experiments, the short hairpin RNA (shRNA)-generating plasmid pSuper/Puro (Oligoengine, Seattle, WA) was used. The oligonucleotide-targeting base sequence for human RasGRF1 was: 5′-GTACCGGAGGATGTCCTTA-3′. RMS cells were plated at 80% confluency and transfected with shRNA vector using Lipofectamine (Invitrogen) according to the manufacturer’s protocol. A commercially available scrambled shRNA negative control plasmid was used (Dharmacon). For stable transfection of shRNA-producing vectors, single-cell dilutions of lipofected cells were prepared and further expansion was performed in the presence of puromycin (1 *μ*g/ml, Invitrogen).

### Cell proliferation

Cells were plated in 24-well culture plates at an initial density of 3×10^3^ cells/well in the presence or absence of IGF-II (100 ng/ml) or insulin (10 ng/ml). In some experiments farnesyl transferase inhibitor FTI277 (Sigma) was used at a concentration of 10 *μ*M. The cell number was calculated at 24, 48, and 72 h after culture initiation. At the indicated time points, cells were harvested from the culture flasks by trypsinization and the number of cells determined using an LSR-II cell cytometer (BD Biosciences).

### Phosphorylation of intracellular pathway proteins and western blotting

Western blots were performed on extracts prepared from RMS cell lines (2×10^6^ cells) that were kept in RPMI medium containing low levels of bovine serum albumin (BSA, 0.5%) to render the cells quiescent. The cells were divided and stimulated with optimal doses of SDF-1 (300 ng/ml), I-TAC (100 ng/ml), HGF (100 ng/ml), IGF-II (100 ng/ml), and insulin (10 ng/ml) for 5 min at 37°C and then lysed (for 10 min) on ice in M-Per lysing buffer (Pierce, Rockford, IL), containing protease and phosphatase inhibitors (Sigma). Subsequently, the extracted proteins were separated by either 12% or 15% sodium dodecyl sulfate-polyacrylamide gel electrophoresis (SDS-PAGE) and the fractionated proteins were transferred to a nitrocellulose membrane (Schleicher & Schuell, Keene, NH) as previously described. RasGRF1 phosphorylated at Ser929 and total RasGRF1 were detected using rabbit polyclonal antibodies (Santa Cruz Biotechnology). Phosphorylation of the intracellular kinases, p42/44 mitogen-activated protein kinase (MAPK) (Thr202/Tyr204) and AKT, was detected using commercial mouse phospho-specific mAb (p42/44) or rabbit phospho-specific polyclonal antibodies (all from New England Biolabs, Beverly, MA) with horseradish peroxidase (HRP)-conjugated goat anti-mouse IgG or goat anti-rabbit IgG as a secondary antibody (Santa Cruz Biotechnology). Equal loading in the lanes was evaluated by stripping the blots and reprobing with appropriate mAbs: p42/44 anti-MAPK clone no. 9102 and anti-AKT clone no. 9272 (Santa Cruz Biotechnology). The membranes were developed with an enhanced chemiluminescence (ECL) reagent (Amersham Life Sciences, Little Chalfont, UK), dried, and exposed to film (HyperFilm, Amersham Life Sciences).

### Ras activity assay

The Ras activation assay kit (Upstate Inc.) was used for these studies according to the manufacturer’s protocol. Cells were serum starved overnight in 0.5% BSA containing RPMI (control) and stimulated with SDF-1 (300 ng/ml) or IGF-II (100 ng/ml) for 5 min and then lysed using an Mg^2+^ lysis buffer (125 mM HEPES, pH 7.5, 750 mM NaCl, 5% NP-40, 50 mM MgCl_2_, 5 mM ethylenediaminetetraacetic acid, and 10% glycerol). Raf-1 Ras-binding domain agarose beads were added to lysates followed by incubation for 1 h at 4°C. Beads were washed twice, and bound Ras-GTP (active form) was detected by immunoblot with a pan-anti-Ras antibody (clone RAS 10). Quantitative analysis of Ras activation bands obtained via western blot analysis was performed using ImageJ software (http://rsb.info.nih.gov/ij/). Relative Ras activity was calculated as percentage of control (−) and corrected for signal intensity with loading controls.

### Chemotaxis assay

The 8-*μ*m Transwell polycarbonate membranes were covered with 50 *μ*l of 0.5% gelatin. Cells were detached with 0.5 mmol/l ethylenediaminetetraacetic acid (EDTA), washed in RPMI-1640, resuspended in RPMI-1640 with 0.5% BSA, and seeded at a density of 3×10^4^ cells in 120 *μ*l into the upper chambers of Transwell inserts (Costar Transwell; Corning Costar, Corning, NY). The lower chambers were filled with SDF-1 (300 ng/ml), I-TAC (100 ng/ml), HGF (100 ng/ml), IGF-II (100 ng/ml), insulin (10 ng/ml), or 0.5% BSA RPMI-1640 (control). After 24 h, the inserts were removed from the Transwell apparatus. Cells remaining in the upper chambers were scraped off with cotton wool and cells that had transmigrated were stained by HEMA 3, according to the manufacturer’s instructions (Fisher Scientific, Pittsburgh, PA), and counted either on the lower side of the membranes or on the bottom of the Transwell inserts.

### Adhesion of RMS cells to fibronectin

Cells were made quiescent for 24 h with 0.5% BSA in RPMI before incubation with SDF-1 (300 ng/ml), I-TAC (100 ng/ml), HGF (100 ng/ml), IGF-II (100 ng/ml), or insulin (10 ng/ml) for 5 min. Cells were added directly onto the protein-coated wells (5×10^3^/well) for 5 min. The wells were coated with fibronectin (10 *μ*g/ml) by incubating overnight at 4°C and blocked with BSA for 2 h before the experiment. Following incubation at 37°C, the plates were vigorously washed 3 times and adherent cells were stained by HEMA 3 and counted under a microscope.

### Fluorescent staining of RMS cells

RMS cells were fixed in 3.5% paraformaldehyde for 20 min, permeabilized by 0.1% Triton X-100, washed in PBS, pre-blocked with 2% BSA and subsequently stained with antibodies to RasGRF1 (1:200, rabbit polyclonal IgG, Santa Cruz, Inc.), paxillin (1:200, mouse monoclonal IgG, eBioscience) and phalloidin - Alexa 488 (1:400, Molecular Probes, Eugene, OR). Appropriate secondary Alexa Fluor 594 mouse anti-rabbit IgG and Alexa Fluor 594 goat anti-mouse IgG were used (1:400, Molecular Probes). The nuclei were identified with DAPI (Molecular Probes). The fluorescence images were collected with the TE-FM Epi-Fluorescence system attached to an Olympus Inverted Microscope IX81 (Olympus, Center Valley, PA).

### Xenografts of human RMS cells into immunodeficient mice

To evaluate the *in vivo* metastatic behavior of three populations of RH30 cells (RH30, RH30 scrambled, and RH30 with knockdown of RasGRF1), cells (5×10^6^ per mouse) were inoculated into the hind limb muscles of SCID/beige inbred mice. Six weeks later, the mice were sacrificed for evaluation of the RMS cells present in blood, bone marrow, liver, and lungs and the presence of RMS cells (i.e., murine-human chimerism) was evaluated by the difference in the level of human α-satellite expression. DNA was amplified in the extracts isolated from bone marrow-, liver- and lung-derived cells using real-time PCR. Briefly, DNA was isolated using the QIAamp DNA Mini kit (Qiagen). Detection of human satellite and murine β-actin DNA levels was conducted by real-time PCR using an ABI PRISM 7500 Sequence Detection System. A 25-*μ*l reaction mixture containing 12.5 *μ*l SYBR Green PCR Master Mix, 300 ng DNA template, and forward (5′-ACC ACT CTG TGT CCT TCG TTG G-3′) and reverse primers (5′-ATC GCG CTC TCA AAA GGA GTG T-3′ and 5′-AAA CGT CCA CTT GCA GAT TCT AG-3′) for the α-satellite sequences and forward (5′-GGA TGC AGA AGG AGA TCA CTG-3′) and reverse primer (5′-CGA TCC ACA CGG AGT ACT TG-3′) for β-actin was used. The Ct value was determined as before. The number of human cells present in the murine organs (indicating the degree of chimerism) was calculated from the standard curve obtained by mixing different numbers of human cells with a constant number of murine cells.

### Statistical analysis

All results are presented as mean ± standard error of the mean (SEM). Statistical analysis of the data was performed using the nonparametric Mann-Whitney test, with p<0.05 considered significant.

## Results

### RasGRF1 is expressed in human RMS cell lines and primary tumors

**Figure 1:**
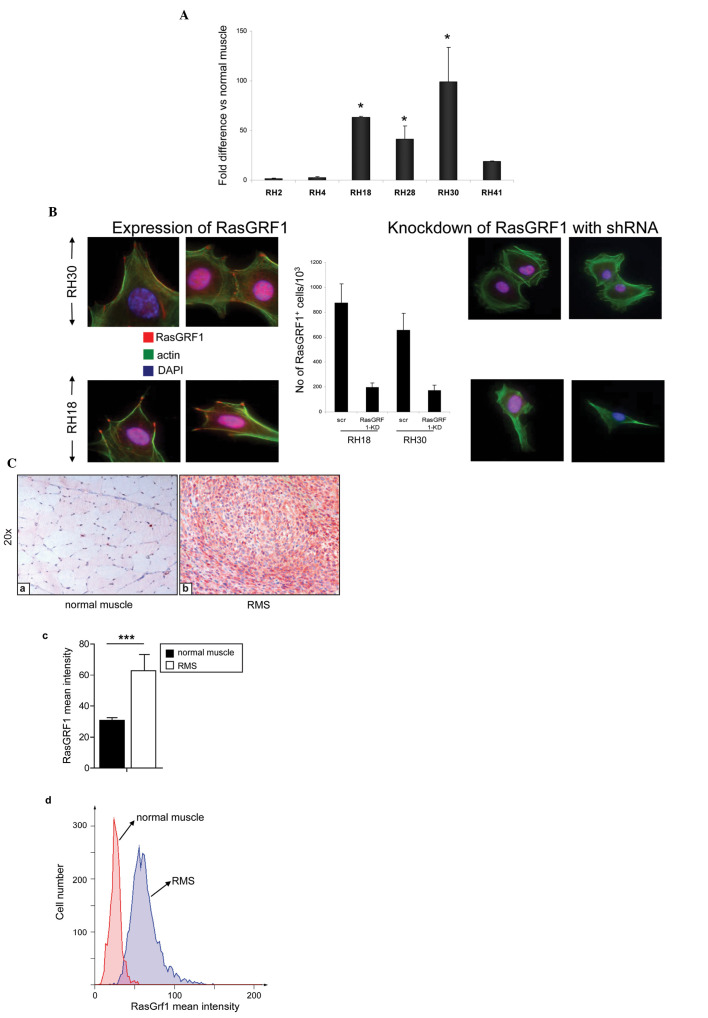
RasGRF1 is overexpressed in human ARMS cell lines and ARMS patient samples. (A), Expression of RasGRF1 in human ARMS cell lines as determined by RQ-PCR. RasGRF1 expression was evaluated by real-time PCR and the fold difference was calculated on the basis of 2ΔCt values normalized by gene expression in normal skeletal muscle, where expression of RasGRF1 in normal muscle tissue is defined as unity. Data from three independent experiments are pooled together. ^*^p<0.05. (B), Expression of RasGRF1 in human ARMS cell lines as determined by immunofluorescent analysis. RasGRF1 was highly expressed in RH30 and RH18 cell lines and diminished expression was observed after treatment with shRNA vector against RasGRF. Representative staining is shown. (C), Expression of RasGFR1 protein in a human normal muscle (a) and ARMS (b). The bar graph (c) shows the significant differences of the RasGRF1 mean intensity of a normal muscle versus ARMS. The data of the mean intensities were generated on samples from 4 ARMS patients with HistoQuest, exported to GraphPad Prism and statistically analyzed. ^*^p<0.0004. (d), The histogram overlay of the normal muscle and RMS. The mean intensity of RMS is much higher compared to the normal muscle. The overlay image was done with HistoQuest.

Fig. 1A shows expression of RasGRF1 mRNA in established human ARMS cell lines (RH2, RH4, RH18, RH28, RH30 and RH41) compared to RasGRF1 mRNA expression in normal human skeletal muscle cells. We observed high (>50 times) RasGRF1 overexpression (at the mRNA level) in 3 and elevated expression in further 2 ARMS (>10 times) out of 6 cell lines. RasGRF1 was also upregulated in 3 out of 3 ERMS cell lines (RD, RH36, SMS-CTR) (data not shown). Interestingly, the ERMS cell line RD transfected with the PAX3-FKHR trans-gene expressed RasGRF1 at several times the level of wild-type RD cells (data not shown). By employing immunofluorescence analysis, we observed that RasGRF1 protein is located in the filopodia of RMS cells (Fig. 1B).

We also evaluated expression of RasGRF1 in human primary ARMS tumor samples and noted pronounced upregulation of its expression at protein level as compared to normal skeletal muscle and surrounding tissue from normal patients (Fig. 1C, data not shown).

### RasGRF1 controls proliferation of human RMS cells

**Figure 2:**
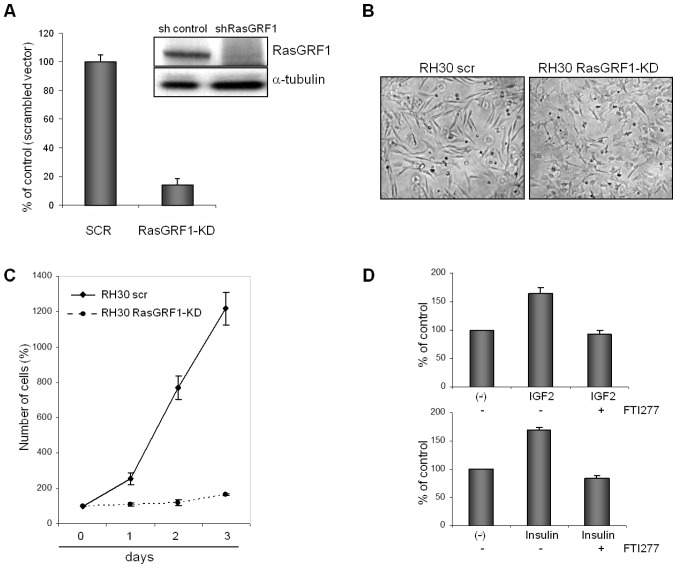
Effect of Ras and RasGRF1 inhibition on ARMS cell proliferation. (A), Effect of RasGRF1 shRNA on mRNA expression. RasGRF1 expression by RQ-PCR in RH30 cells transfected with scrambled shRNA vector or RH30 cells transfected with a shRNA vector down-regulating RasGRF1 expression (RH30 RasGRF1-kd cells). (B), Changes in cell morphology after down-regulating RasGRF1 expression. Light micrograph (magnification ×10) showing difference in cell morphology between RH30 cells transfected with scrambled control vector (RH30scr) and cells transfected with shRNA vector down-regulating RasGRF1 expression (RH30 RasGRF1-kd). (C), Kinetics of growth of RH30 cell line after down-regulation of RasGRF1 expression. Similarly to previous experiments, two RH30-derived cell lines were used (RH30scr and RH30 RasGRF1-kd). Cells were grown for 72 h in RPMI supplemented with 10% FBS. Data averaged from three independent experiments are shown. (D), Effect of Ras protein inhibition by farnesyl transferase inhibitor (FTI277). RH30scr cells were grown for 72 h in serum-depleted medium (RPMI + 0.5% BSA) (−), supplemented with either 200 ng/ml IGF-II or 10 ng/ml insulin with the addition of 10 *μ*M FTI277. The experiment was repeated twice.

In further experiments, we selected the ARMS cell line RH30 that expresses RasGRF1 at the highest level (Fig. 1A) and, as we reported previously, responds robustly to several pro-metastatic chemoattractants such as SDF-1 (30), HGF/SF (31), and I-TAC (32). Fig. 2A and Fig. 1B show that we were able to use an shRNA technology to efficiently down-regulate expression of RasGRF1 in RH30 cells both at the mRNA (Fig. 2A) and protein levels (Fig. 1B). RH30 cells with down-regulated RasGRF1 changed morphology from a spindle-forming to a more flat phenotype (Fig. 2B). Most importantly, down-regulation of RasGRF1 expression in RH30 cells resulted in a decrease in proliferative potential (Fig. 2C). These cells however, were still alive and did not undergo apoptosis as evaluated by 0.4% trypan blue exclusion test and Annexin-V staining.

In parallel, we also knocked down expression of RasGRF1 in the RH18 cell line, which similarly to RH30, also highly expresses RasGRF1 and obtained similar results (data not shown).

Igf-2 and Ins are known factors that stimulate proliferation of RMS cells (10–12). Thus, we stimulated RH30 cells by Igf-2 or Ins in the presence or absence of the Ras-GTPase blocking agent farnesyl transferase inhibitor (FTI 277). As shown in Fig. 2D, the pro-proliferative effect of Igf-2 and Ins was inhibited in the presence of FTI 277.

### RasGRF1 is involved in intracellular signaling after stimulation by pro-metastatic chemoattractants

**Figure 3:**
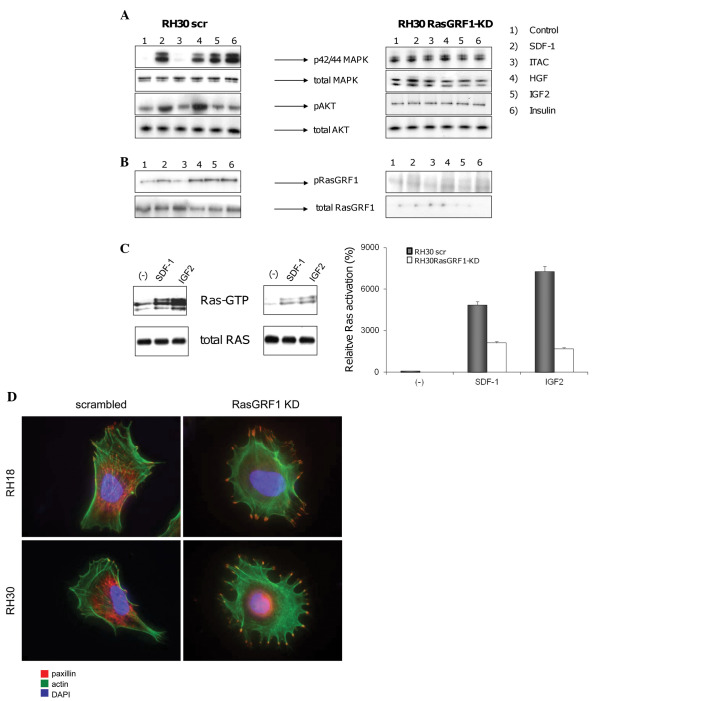
RasGRF1 is involved in chemokine and growth factor receptor signaling. (A), Effect of RasGRF1 down-regulation on activation of intracellular signaling in ARMS cells. Phosphorylation of p42/44 MAPK and AKT in RH30-derived cell lines was stimulated for 5 min by SDF-1 (300 ng/ml), I-TAC (100 ng/ml), HGF (100 ng/ml), IGF-II (100 ng/ml), and insulin (10 ng/ml). The experiment was repeated three times with similar results. A representative result is shown. (B), RasGRF1 phosphorylation after stimulation with chemokines and growth factors. RasGRF1 protein phophorylated at Ser929 was detected by western blot analysis after stimulation for 5 min by SDF-1 (300 ng/ml), I-TAC (100 ng/ml), HGF (100 ng/ml), IGF-II (100 ng/ml), and insulin (10 ng/ml). (C), Effect of RasGRF1 down-regulation on Ras activation. A Ras pull-down assay was performed on two RH30-derived cell lines (RH30scr and RH30 RasGRF1-kd). The cells were stimulated for 5 min with SDF-1 (300 ng/ml) or IGF-II (100 ng/ml). Ras-GTP was precipitated by Raf-1 RBD agarose conjugate and detected by Ras antibody clone RAS10 (Millipore). The same antibody was used to detect total Ras protein (p21 H-, K- and N-Ras). Western blots were analyzed by densitometry (right side). The experiment was repeated three times with similar results. A representative result is shown. (D), Effect of RasGRF1 down-regulation on paxillin expression and actin cytoskeleton. Staining of paxillin and actin was performed on RH30scr, RH30 RasGRF1-kd, RH18scr and RH18 RasGRF-kd cell lines. The experiment was repeated three times and representative results are shown.

As reported (9–11, 30–33) the pro-metastatic behavior of RMS cells is influenced by several chemoattractants/growth factors (i.e., SDF-1, I-TAC, HGF/SF, Igf-2, and Ins). Therefore, in the next step we evaluated phosphorylation of RasGRF1 in RH30 wild-type (RH30 wt) and RH30 RasGRF1-kd cells (Fig. 3A). In RH30 wt cells we observed an increase in both p42/44 MAPK and AKT phosphorylation after stimulation by SDF-1 and HGF/SF and an increase in p42/44 MAPK phosphorylation alone after stimulation by Igf-2 and Ins. Since RH30 cells express very low levels of CXCR7 (32), no activation/phosphorylation of p42/44 MAPK and/or AKT was observed when the CXCR7 ligand I-TAC was employed for stimulation. In striking contrast, no phosphorylation was detected in RH30 RasGRF1-kd cells. Fig. 3B confirms that stimulation by SDF-1, HGF/SF, Igf-2, and Ins activates phosphorylation of RasGRF1 in RH30 wt but it was not detectable in RH30 RasGRF1-kd cells.

These data *in toto* demonstrate that RasGRF1 is required for signaling from the SDF-1 receptor (CXCR4), the HGF receptor (c-met), the Igf-2 receptor (IGF-2R) and the Ins receptor (INS-R). The Ras GTP pull-down assay data shown in Fig. 3C confirm that Ras is activated in response to SDF-1 and Igf-2 in RH30 wt cells, but as expected, is activated at only very low levels in RH30 RasGRF1-kd cells.

To address the defect in migration of RasGRF1-kd cells we evaluated by confocal microscope the distribution of paxillin, that is involved in interaction between β-integrins on cell surface and kinases, tructural proteins and regulators of actin organization in cytoplasm. As predicted from their less migratory phenotype, RasGRF1-kd cells show a high accumulation of paxillin in filopodia and more adherent phenotype as compared to control cells (Fig. 3D).

### RasGRF1 is involved in migration but not adhesion of RMS cells

**Figure 4:**
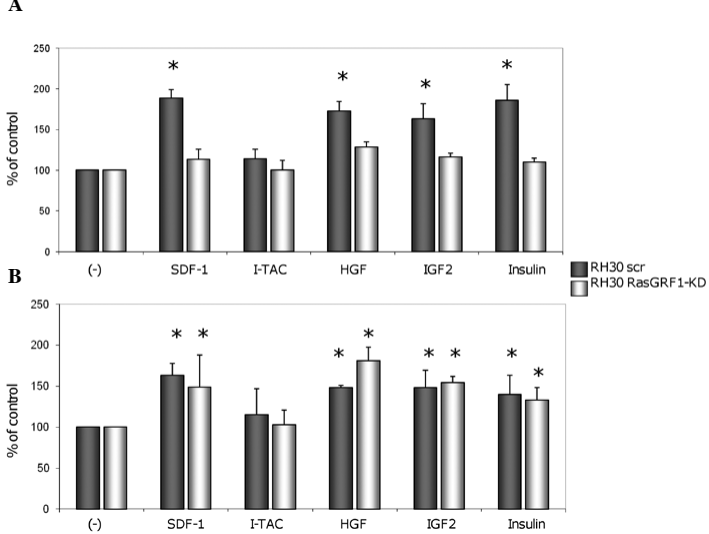
Effect of RasGRF1 knockdown on migration and adhesion of ARMS cells. (A), ARMS cell chemotaxis after down-regulating RasGRF1 expression. Chemotaxis of RH30-derived cells across Transwell membranes covered with gelatin in response to SDF-1 (300 ng/ml), I-TAC (100 ng/ml), HGF (100 ng/ml), IGF-II (100 ng/ml), and insulin (10 ng/ml) gradients. Black bars show chemotaxis of control RH30scr cells, while white bars represent chemotaxis of RH30 cells with down-regulated expression of RasGRF1. Data from 4 separate experiments are pooled together. ^*^p<0.05 compared to unstimulated controls (−). (B), ARMS cell adhesion after down-regulating RasGRF1 expression. Adhesion of human RMS cells to fibronectin after stimulation by SDF-1 (300 ng/ml), I-TAC (100 ng/ml), HGF (100 ng/ml), IGF-II (100 ng/ml), and insulin (10 ng/ml). Data from 3 separate experiments are pooled together. ^*^p<0.05 as compared to unstimulated controls (−).

Next we evaluated the responsiveness of RH30 wt and RH30 RasGRF1-kd cells to selected chemoattractants in chemotaxis (Fig. 4A) and adhesion assays (Fig. 4B). We observed that down-regulation of RasGRF1 in RH30 cells resulted in inhibition of responsiveness of these cells to chemotactic gradients of SDF-1, HGF/SF, Igf-2, and insulin. However, this down-regulation did not significantly decrease the adhesive potential of these cells in response to the same factors. In control experiments, inhibition of chemotaxis was also observed when we blocked Ras-GTPase by employing farnesyl transferase inhibitor (FTI 277) (data not shown).

### Effect of RasGRF1 down-regulation on in vivo tumor growth of RH30 cells

**Figure 5:**
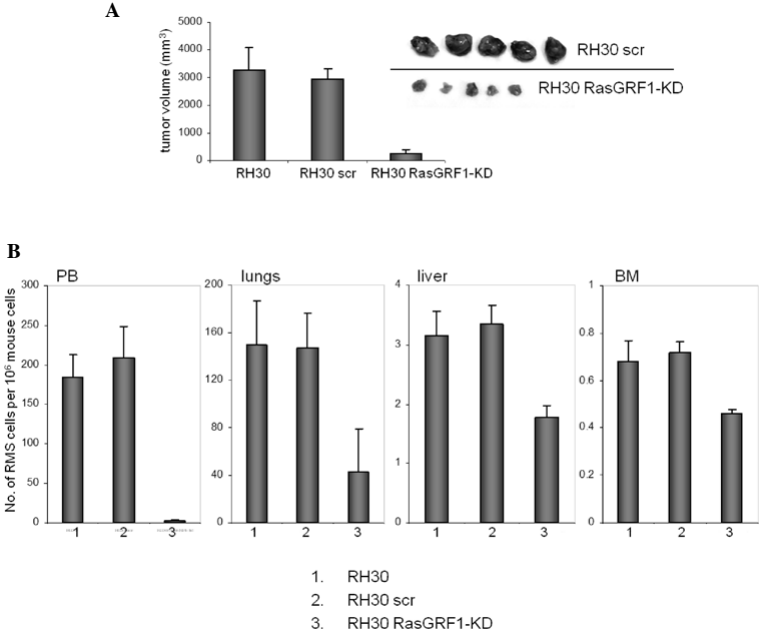
Down-regulation of RasGRF1 inhibits ARMS cell growth *in vivo* and metastasis. (A), ARMS tumor formation after down-regulating RasGRF1 expression. Tumor formation by RH30 wild-type, RH30scr, and RH30 RasGRF1-kd cells inoculated into the hind limb muscles of SCID/Beige inbred mice. Six weeks later, mice were sacrificed and femora were harvested to evaluate the size of the growing tumor. In the experiment five mice were used for each RH30-derived cell line. *p<0.001. (B), Detection of human cells in organs. Additionally, peripheral blood, livers, bone marrows, and lungs were harvested from mice inoculated with RH30 and RH30-derived cells and the human RMS cells were detected by RQ-PCR. *p<0.05.

Finally, we employed SCID/beige mice to study tumor formation by RH30 wt and RH30 RasGRF1-kd cells (Fig. 5). We observed that after down-regulation of RasGRF1, RH30 cells formed significantly smaller tumors following inoculation into skeletal muscles of immunodeficient SCID/beige mice (Fig. 5A).

Furthermore, 6 weeks after inoculation of RMS cells, we observed a much lower number of RMS cells in peripheral blood, lungs, liver, and bone marrow of mice inoculated with RasGRF1-kd RH30 cells (Fig. 5B).

## Discussion

RMS is the most common soft tissue sarcoma in children that, as recently postulated, originates from mutated primitive mesodermal/mesenchymal stem cells (ARMS) or skeletal muscle satellite cells (ERMS) (8). Our recent research on VSELs, which are deposited during development in tissues of young individuals and whose number rapidly declines with the age, allowed us to present the hypothesis that VSELs are a ‘missing link’ that would reconcile the embryonic rest/germ line origin of cancer postulated 150 years ago with contemporary theories of cancer development (34). In support of this, the analysis of epigenetic changes in VSELs identified unique methylation patterns of DMRs in some imprinted genes (Igf2-H19, KCNQ1 and RasGRF1) that, on the one hand, explain the dormant state of VSELs residing in adult tissues (27) but on the other hand, explain the reverse pattern of expression of these genes reported in RMS seen for example in Beckwith-Wiedemann syndrome patients (35–38). Since as recently demonstrated VSELs are at the top of the hierarchy of the mesenchymal stem cell lineage (39), changes in the epigenetic state of DMR in some of the imprinted genes in VSELs or VSELs-derived mesenchymal stem cells could potentially trigger RMS development.

In research leading to the current paper we became interested in events downstream from activated receptors and focused on RasGRF1, which is a GEF for the Ras superfamily of GTPases and is paternally imprinted in mice (40–42). While Ras proteins regulate various signaling pathways controlling cell growth, differentiation, and survival, RasGRF1 was initially described as highly expressed in brain tissue, while playing a role in learning and memory. The full-length RasGRF1 protein contains several domains: a pleckstrin homology domain, a coiled-coil region, a calmodulin-dependent activation domain, the ilimaquinon motif, a DBL homology domain, and a CDC25 domain (43,44). Alternative forms of RasGRF1, ranging in size from approximately 50 to 140 kDa, have been identified and overexpression of larger forms has been reported to be crucial for transformation of NIH 3T3 cells (45). Interestingly, the p75 isoform has been reported to be a more effective GEF for H-Ras (46).

It has been reported that RasGRF1 knockout mice are smaller than normal littermates and display defects in memory consolidation associated with different areas of the brain, as well as defects in β-cell development and glucose homeostasis (47). It has been demonstrated that RasGRF1 is a c-Jun-regulated gene necessary for promoting non-adherent growth of c-Myc- or c-Jun-transduced fibroblasts and not much attention has been paid to a potential role of RasGRF1 in tumorigenesis, despite this protein having been observed to be expressed in several tumor types (48).

As demonstrated in this study, RasGRF1, compared to normal skeletal muscles, is overexpressed in the majority of RMS cell lines, which was subsequently confirmed by immunohistochemical detection in patient samples. However, since we compared RasGRF1 expression in proliferating ARMS cells to normal skeletal muscles, further studies are needed to see if this phenomenon is a result of malignant phenotype of ARMS cells or rather depends on proliferative status of myogenic cells.

Moreover, we observed that RasGRF1 becomes phosphorylated/activated after stimulation with prometastatic factors, such as SDF-1 and HGF/SF, which suggests its involvement in signaling of the CXCR4 and c-met receptors, respectively. We also confirmed our previous observations that in RH30 cells, SDF-1 and HGF/SF activate p42/44 MAPK and AKT, which are involved in cell migration (30,31). The potential involvement of RasGRF1 in RMS cell migration was subsequently confirmed by confocal microscopy observations that this GEF localizes within cell filopodia. More importantly, we noticed that knockdown of RasGRF1 in ARMS cells abolished their chemotactic responsiveness to several prometastatic factors. This inhibition correlated with a lack of activation of p42/44 MAPK and AKT, which suggests that RasGRF1 acts downstream of, for example, the CXCR4 and c-met receptors involved in cell migration.

Interestingly, the knockdown of RasGRF1 did not affect adhesion of ARMS cells. Compared to wild-type cells, ARMS RasGRF1-kd cells showed normal adhesion and microscopic evaluation of these cells revealed that they change from a spindle-forming to a more flat morphology. Interestingly, we noted in RasGRF1-kd cells accumulation in filopodia of paxillin that is involved in interaction between β-integrins on cell surface and kinases, structural proteins and regulators of actin organization in cytoplasm, which supports our data that down-regulation of RasGRF1 inhibits RMS cells migration but does not affect their adhesion.

It has been reported that insulin family factors strongly stimulate proliferation of RMS cells, and Igf-2 is an autocrine factor interacting with both Igf-1R and Ins-R. In this study we report that knockdown of RasGRF1 by shRNA and inhibition of Ras by FTI277 results in inhibition of ARMS cell proliferation. More importantly, RasGRF1-kd cells inoculated in immunodeficient Beige-SCID mice formed significantly smaller tumors. In addition, we observed a significantly lower number of circulating ARMS cells in peripheral blood in these animals compared to mice bearing tumors formed by control RasGRF1-scr or wt cells. Of note, compared to wt cells, RasGRF1-kd cells did not respond by chemotaxis to Igf-2 or an Ins gradient, which correlated with a lack of activation of p42/44 MAPK and AKT in these cells by these factors.

Overall, our data suggest that RasGRF1 is required for Ras-mediated ARMS proliferation. The involvement of RasGRF1 in insulin factor-mediated cell proliferation is supported by recent observations that both RasGRF1 knockout mice as well as bimaternal mice, which in the first 10 weeks after birth display defective RasGRF1 expression, have reduced body size (47). Furthermore, as reported, Igf-1 did not stimulate proliferation of β-cells from RasGRF1-deficient mice and did not activate p42/44 MAPK and AKT in these cells. All these observations could be explained by involvement of RasGRF1 in insulin/insulin-like growth factor signaling, and our signal transduction data lend support to this hypothesis. However, we are aware that since we compared RasGRF1 expression in proliferating ARMS cells to normal non-dividing skeletal muscles, further studies are needed to see if this phenomenon is the result of malignant phenotype of ARMS cells or rather depends on cell proliferation.

Since RasGRF1 can act also as GEF for Rac, further studies will answer which of these RasGRF1 functions are related to Rac activation (14). Similar studies are required to shed more light on the role of RasGRF1-interacting partner proteins that were recently identified by large-scale proteomic analysis, including ribosomal and RNA-binding proteins, cytoskeletal proteins, and some other proteins involved in vesicular trafficking (15,49,50).

In summary, our data for the first time demonstrate a role for RasGRF1 in signaling from CXCR4, c-met, Igf-1R, and Ins-R receptors, which is crucial for ARMS migration, metastasis, and growth. We conclude that RasGRF1, which plays an important role in ARMS pathogenesis, is a new potential target to develop efficient, small, blocking molecules to inhibit RMS growth.

## References

[1] Barr FG, Galili N, Holick J, Biegel JA, Rovera G, Emanuel BS (1993). Rearrangement of the PAX3 paired box gene in the paediatric solid tumour alveolar rhabdomyosarcoma. Nat Genet.

[2] Collins MH, Zhao H, Womer RB, Barr FG (2001). Proliferative and apoptotic differences between alveolar rhabdomyosarcoma subtypes: a comparative study of tumors containing PAX3-FKHR or PAX7-FKHR gene fusions. Med Pediatr Oncol.

[3] Hazelton BJ, Houghton JA, Parham DM (1987). Characterization of cell lines derived from xenografts of childhood rhabdomyosarcoma. Cancer Res.

[4] Kelly KM, Womer RB, Barr FG (1998). PAX3-FKHR and PAX7-FKHR gene fusions in rhabdomyosarcoma. J Pediatr Hematol Oncol.

[5] Sandberg AA, Stone JF, Czarnecki L, Cohen JD (2001). Hematologic masquerade of rhabdomyosarcoma. Am J Hematol.

[6] Sharp R, Recio JA, Jhappan C (2002). Synergism between INK4a/ARF inactivation and aberrant HGF/SF signaling in rhabdomyosarcomagenesis. Nat Med.

[7] Gordon T, McManus A, Anderson J (2001). Cytogenetic abnormalities in 42 rhabdomyosarcoma: a United Kingdom Cancer Cytogenetics Group Study. Med Pediatr Oncol.

[8] Charytonowicz E, Cordon-Cardo C, Matushansky I, Ziman M (2009). Alveolar rhabdomyosarcoma: is the cell of origin a mesenchymal stem cell?. Cancer Lett.

[9] Hahn H, Wojnowski L, Specht K (2000). Patched target Igf2 is indispensable for the formation of medulloblastoma and rhabdomyosarcoma. J Biol Chem.

[10] Makawita S, Ho M, Durbin AD, Thorner PS, Malkin D, Somers GR (2009). Expression of insulin-like growth factor pathway proteins in rhabdomyosarcoma: IGF-2 expression is associated with translocation-negative tumors. Pediatr Dev Pathol.

[11] Rikhof B, De Jong S, Suurmeijer AJ, Meijer C, van der Graaf WT (2009). The insulin-like growth factor system and sarcomas. J Pathol.

[12] Wang W, Kumar P, Epstein J, Helman L, Moore JV, Kumar S (1998). Insulin-like growth factor II and PAX3-FKHR cooperate in the oncogenesis of rhabdomyosarcoma. Cancer Res.

[13] Naini S, Etheridge KT, Adam SJ (2008). Defining the cooperative genetic changes that temporally drive alveolar rhabdomyosarcoma. Cancer Res.

[14] Innocenti M, Zippel R, Brambilla R, Sturani E (1999). CDC25(Mm)/Ras-GRF1 regulates both Ras and Rac signaling pathways. FEBS Lett.

[15] Lavagni P, Indrigo M, Colombo G (2009). Identification of novel RasGRF1 interacting partners by large-scale proteomic analysis. J Mol Neurosci.

[16] Rossman KL, Der CJ, Sondek J (2005). GEF means go: turning on RHO GTPases with guanine nucleotide-exchange factors. Nat Rev Mol Cell Biol.

[17] Langenau DM, Keefe MD, Storer NY (2007). Effects of RAS on the genesis of embryonal rhabdomyosarcoma. Genes Dev.

[18] Shome D, Honavar SG, Reddy VA, Vemuganti GK (2007). Orbital embryonal rhabdomyosarcoma in association with neurofibromatosis type 1. Ophthal Plast Reconstr Surg.

[19] Yang P, Grufferman S, Khoury MJ (1995). Association of childhood rhabdomyosarcoma with neurofibromatosis type I and birth defects. Genet Epidemiol.

[20] Jung A, Bechthold S, Pfluger T, Renner C, Ehrt O (2003). Orbital rhabdomyosarcoma in Noonan syndrome. J Pediatr Hematol Oncol.

[21] Khan S, McDowell H, Upadhyaya M, Fryer A (1995). Vaginal rhabdomyosarcoma in a patient with Noonan syndrome. J Med Genet.

[22] Gripp KW, Scott CI, Nicholson L (2002). Five additional Costello syndrome patients with rhabdomyosarcoma: proposal for a tumor screening protocol. Am J Med Genet.

[23] Feingold M (1999). Costello syndrome and rhabdomyosarcoma. J Med Genet.

[24] O’Neal JP, Ramdas J, Wood WE, Pellitteri PK (2004). Parameningeal rhabdomyosarcoma in a patient with Costello syndrome. J Pediatr Hematol Oncol.

[25] Tidyman WE, Rauen KA (2009). The RASopathies: developmental syndromes of Ras/MAPK pathway dysregulation. Curr Opin Genet Dev.

[26] Hettmer S, Wagers AJ (2010). Muscling in: uncovering the origins of rhabdomyosarcoma. Nat Med.

[27] Shin DM, Zuba-Surma EK, Wu W (2009). Novel epigenetic mechanisms that control pluripotency and quiescence of adult bone marrow-derived Oct4(+) very small embryonic-like stem cells. Leukemia.

[28] Stratton MR, Fisher C, Gusterson BA, Cooper CS (1989). Detection of point mutations in N-ras and K-ras genes of human embryonal rhabdomyosarcomas using oligonucleotide probes and the polymerase chain reaction. Cancer Res.

[29] Ren YX, Finckenstein FG, Abdueva DA (2008). Mouse mesenchymal stem cells expressing PAX-FKHR form alveolar rhabdomyosarcomas by cooperating with secondary mutations. Cancer Res.

[30] Libura J, Drukala J, Majka M (2002). CXCR4-SDF-1 signaling is active in rhabdomyosarcoma cells and regulates locomotion, chemotaxis, and adhesion. Blood.

[31] Jankowski K, Kucia M, Wysoczynski M (2003). Both hepatocyte growth factor (HGF) and stromal-derived factor-1 regulate the metastatic behavior of human rhabdomyosarcoma cells, but only HGF enhances their resistance to radiochemotherapy. Cancer Res.

[32] Grymula K, Tarnowski M, Wysoczynski M (2010). Overlapping and distinct role of CXCR7-SDF-1/ITAC and CXCR4-SDF-1 axes in regulating metastatic behavior of human rhabdomyosarcomas. Int J Cancer.

[33] Tarnowski M, Grymula K, Reca R (2010). Regulation of expression of stromal-derived factor-1 receptors: CXCR4 and CXCR7 in human rhabdomyosarcomas. Mol Cancer Res.

[34] Ratajczak MZ, Shin DM, Liu R (2010). Epiblast/germ line hypothesis of cancer development revisited: lesson from the presence of Oct-4^+^ cells in adult tissues. Stem Cell Rev.

[35] Casola S, Pedone PV, Cavazzana AO (1997). Expression and parental imprinting of the H19 gene in human rhabdomyosarcoma. Oncogene.

[36] Anderson J, Gordon A, McManus A, Shipley J, Pritchard-Jones K (1999). Disruption of imprinted genes at chromosome region 11p15.5 in paediatric rhabdomyosarcoma. Neoplasia.

[37] Scrable H, Cavenee W, Ghavimi F, Lovell M, Morgan K, Sapienza C (1989). A model for embryonal rhabdomyosarcoma tumorigenesis that involves genome imprinting. Proc Natl Acad Sci USA.

[38] Zhan S, Shapiro DN, Helman LJ (1994). Activation of an imprinted allele of the insulin-like growth factor II gene implicated in rhabdomyosarcoma. J Clin Invest.

[39] Taichman RS, Wang Z, Shiozawa Y (2010). Prospective identification and skeletal localization of cells capable of multilineage differentiation in vivo. Stem Cells Dev.

[40] De la Puente A, Hall J, Wu YZ (2002). Structural characterization of Rasgrf1 and a novel linked imprinted locus. Gene.

[41] Yoon B, Herman H, Hu B (2005). Rasgrf1 imprinting is regulated by a CTCF-dependent methylation-sensitive enhancer blocker. Mol Cell Biol.

[42] Yoon BJ, Herman H, Sikora A, Smith LT, Plass C, Soloway PD (2002). Regulation of DNA methylation of Rasgrf1. Nat Genet.

[43] Martegani E, Vanoni M, Zippel R (1992). Cloning by functional complementation of a mouse cDNA encoding a homologue of CDC25, a Saccharomyces cerevisiae RAS activator. EMBO J.

[44] Buchsbaum R, Telliez JB, Goonesekera S, Feig LA (1996). The N-terminal pleckstrin, coiled-coil, and IQ domains of the exchange factor Ras-GRF act cooperatively to facilitate activation by calcium. Mol Cell Biol.

[45] Chevallier-Multon MC, Schweighoffer F, Barlat I (1993). Saccharomyces cerevisiae CDC25 (1028–1589) is a guanine nucleotide releasing factor for mammalian ras proteins and is oncogenic in NIH3T3 cells. J Biol Chem.

[46] Leaner VD, Donninger H, Ellis CA, Clark GJ, Birrer MJ (2005). p75-Ras-GRF1 is a c-Jun/AP-1 target protein: its up regulation results in increased Ras activity and is necessary for c-Jun-induced nonadherent growth of Rat1a cells. Mol Cell Biol.

[47] Font de Mora J, Esteban LM, Burks DJ (2003). Ras-GRF1 signaling is required for normal beta-cell development and glucose homeostasis. EMBO J.

[48] Guerrero C, Rojas JM, Chedid M (1996). Expression of alternative forms of Ras exchange factors GRF and SOS1 in different human tissues and cell lines. Oncogene.

[49] Forlani G, Baldassa S, Lavagni P, Sturani E, Zippel R (2006). The guanine nucleotide exchange factor RasGRF1 directly binds microtubules via DHPH2-mediated interaction. FEBS J.

[50] Arozarena I, Matallanas D, Berciano MT (2004). Activation of H-Ras in the endoplasmic reticulum by the RasGRF family guanine nucleotide exchange factors. Mol Cell Biol.

